# Periprosthetic joint infections in a city hospital in Türkiye: A cross-sectional study

**DOI:** 10.1097/MD.0000000000045783

**Published:** 2026-05-12

**Authors:** Bülent Kaya, Suzan Şahin, Serap Demir Tekol, Engin Eceviz

**Affiliations:** a Department of Infection Disease, Kartal Dr Lütfi Kirdar City Hospital, Kartal, Türkiye; b Department of Microbiology, Kartal Dr Lütfi Kirdar City Hospital, Kartal, Türkiye; c Department of Orthopaedics and Traumatology, Kartal Dr Lütfi Kirdar City Hospital, Kartal, Türkiye.

**Keywords:** periprosthetic joint infections, total hip arthroplasty, total knee arthroplasty

## Abstract

Increased life expectancy increases the risk of osteoarthritis and sustaining fractures. Prosthetic surgery is the primary treatment for such conditions. Periprosthetic joint infections (PJI) are the principal cause of failure of arthroplasty. This study aimed to mitigate the incidence of PJI by identifying the predominant risk factors and causative microorganisms associated with PJI at our institution. Surgeries for total hip arthroplasty (THA) and total knee arthroplasty (TKA) conducted at the Orthopedic and Traumatology Clinic were retrospectively reviewed. Patient’ demographic data, reproductive factors, and antibiotic resistance profiles were evaluated. Categorical variables are expressed as frequencies and percentages, while continuous variables with non-normal distributions are presented as median values. Within a span of 7 years, 300 THA and TKA procedures were performed, resulting in 18 patients developing surgical site infections attributed to 24 discrete factors. Of these patients, 15 (83.3%) were female and 3 (16.7%) were male. The age range of the affected individuals was 23 to 93 years, with a median age of 74 years. Sixteen patients underwent THA (89%), whereas the others underwent TKA. The mean duration of hospitalization was 46 days. The predominant risk factors identified were advanced age, female sex, and use of peripheral venous catheters. Microbial cultures revealed an 83.4% prevalence of Gram-negative bacteria and a 16.6% prevalence of Gram-positive bacteria. *Escherichia coli* and *Acinetobacter baumannii* were the most frequently isolated microorganisms. PJIs present a diagnostic challenge and often require prolonged treatment. Importantly, the prevention of PJIs is not only more straightforward but also significantly more cost-effective than treatment. Establishing laminar airflow in the operating room, limiting personnel traffic, implementing institution-specific infection prevention protocols, and providing feedback to staff can reduce the incidence of PJIs. All procedures performed in studies involving human participants were in accordance with the ethical standards of the institutional and/or national research committee and with the 1964 Helsinki Declaration and its later amendments or comparable ethical standards.

## 1. Introduction

The increase in life expectancy of the general population has led to an increase in chronic diseases. Consequently, the risk of osteoarthritis (OA) and fractures in weight-bearing joints, such as the hip and knee, increase with age.^[1,2]^ OA, a degenerative joint disease, typically causes localized pain and movement limitations due to deterioration and volume loss of cartilage stemming from chronic inflammation. Predominantly afflicting individuals in the middle and older age groups, OA significantly impairs quality of life. Currently, the optimal treatment to alleviate pain and restore joint function is an artificial joint prosthesis.^[3,4]^ Infections are one of the main causes of joint arthroplasty.^[1,2,4]^ This study aimed to identify the risk factors, causative microorganisms, and preventive strategies associated with periprosthetic joint infections (PJI) in total hip arthroplasty (THA) and total knee arthroplasty (TKA) performed at our clinic.

## 2. Materials and methods

Infections related to TKA and THA, which necessitated hospitalization and follow-up at our hospital’s Orthopedics and Traumatology Clinic, were retrospectively reviewed via the hospital’s automation system for a period of 7 years, from September 2016 to August 2023. Among the 816 patients aged ≥ 18 years who underwent prosthetic surgeries, 300 underwent TKA and THA. This study excluded patient who underwent other prosthetic procedures. Eighteen patients developed a PJI. While most studies on this subject are multicenter or meta-analytical in nature, our study was designed as a single-center cross-sectional analysis; therefore, the required sample size could not be attained. There was no risk of bias in our study and the findings were entirely reliable. To enhance the statistical strength and reliability of the study, it is necessary to increase the sample size. Infections arising within the first 3 months post-surgery were classified as early PJI, those developing between 3 and 12 months as delayed PJI, and those manifesting after 12 months as late PJI.^[5,6]^ In this study, 24 isolated microorganisms were used. We determined the risk factors that the patients were exposed to, concurrent diseases, causative microorganisms, and antibiotic resistance patterns.

### 2.1. Ethical approval

This study received ethical approval from the Clinical Research Ethics Committee at the University of Health Sciences’ Kartal Dr Lütfi Kirdar City Hospital on September 27, 2023, under decision number 2023/514/258/35.

The analysis and reporting of the results complied with the Strengthening the Reporting of Observational Studies in Epidemiology (STROBE) checklist.

### 2.2. Statistical analysis

Owing to the limited number of cases, this study was designed as a retrospective, observational, and interpretative analysis. Categorical variables are presented as frequencies and percentages, while continuous variables that did not follow a normal distribution are reported as median values.

## 3. Results

PJI developed in 18 (6%) of the 300 patients who underwent TKA and THA. Of these patients, 15 (83.3%) were female and 3 (16.7%) were male. The age range of the affected individuals was 23 to 93 years, with a median age of 74 years. The duration of hospitalization varied from 15 to 92 days, with a median of 46 days. A total of 24 microorganisms were isolated from 18 infected patients.

Advanced age was observed in 17 (94.4%) patients. The presence of a peripheral venous catheter was observed in 15 patients (83.3%). Resistance at the operation site was identified in 9 patients (37.5%). Transfusion of erythrocyte suspension and use of H2 receptor antagonists were reported in 5 (20.8%) patients. A urinary catheter and a central venous catheter were present in 4 (16.7%). One patient (4.2%) required a pericardial tube, and postoperative intubation in the intensive care unit was necessary for another patient.

Of the patients studied, 10 (55.6%) had hypertension and 5 (27.8%) had diabetes mellitus (DM). The accompanying diseases are shown in Table 1.

**Table 1:** Accompanying diseases.

Diseases	n	%
Hypertension	10	55.6
Diabetes mellitus	5	27.8
Anemia	3	16.7
Dementia	3	16.7
Congestive heart failure	3	16.7
Osteoarthritis	2	11.1
Chronic kidney disease	2	11.1
Cerebrovascular disease	2	11.1
Rheumatoid arthritis	2	11.1
Atrial fibrillation	2	11.1
Coronary artery disease	2	11.1
Chronic obstructive pulmonary disease	2	11.1
Gout	1	5.6
Parkinson disease	1	5.6
Renal transplantation	1	5.6
Hyperthyroidism	1	5.6
Chronic liver disease	1	5.6
Asthma bronchiale	1	5.6
Ventricular fibrillation	1	5.6
Tricuspid regurgitation	1	5.6
Myelodysplastic syndrome	1	5.6
Delirium	1	5.6
Benign prostatic hypertrophy	1	5.6
Acute myeloid leukemia	1	5.6

All cases involved early PJIs, with 16 occurring after THA and 2 following TKA. Regarding the site of infection, 16 (66.7%) were diagnosed with primary deep incisional SIIs, 7 (29.2%) with PJI, and 1 (4.1%) with primary superficial incisional SII (Table 2).

**Table 2:** Classification of surgical site infections.

	n	%
Primary deep incisional SSI	16	66.7
PJIs	7	29.2
Primer superficial incisional SSI	1	4.1

PJIs = periprosthetic joint infections, SSI = surgical site infections.

Among the cultured microorganisms, 83.4% (n = 20) were Gram-negative, with *Escherichia coli* constituting 29.2%, *Acinetobacter baumannii* 20.9*%*, *Enterobacter cloacae* 12.5%, *Klebsiella pneumonia*, and *Pseudomonas aeruginosa* each 8.3%, *and Morganella morganii* 4.2%. Gram-positive agents accounted for 16.6% (n = 4), with *Enterococcus faecalis* and *Staphylococcus aureus* isolated in 8.3% of the cases (Table 3). The *A baumannii* strains demonstrated sensitivity to tigecycline and colistin but exhibited resistance to all other tested antibiotics. All Gram-negative agents, excluding *E cloacae*, showed no resistance to carbapenems; however, they exhibit significant resistance to other antibiotic classes. Notably, carbapenem resistance was pronounced in *E cloacae* strains. Both the *E faecalis* strains were sensitive to ampicillin. Among the *S aureus* strains, one showed methicillin resistance (Table 4).

**Table 3:** Rates of breeding microorganisms.

Microorganism	n	%
*Escherichia coli*	7	29.2
*Acinetobacter baumannii*	5	20.9
*Enterobacter cloacae*	3	12.5
*Klebsiella pneumoniae*	2	8.3
*Pseudomonas aeruginosa*	2	8.3
*Enterococcus faecalis*	2	8.3
*Staphylococcus aureus*	2	8.3
*Morganella morganii*	1	4.2

**Table 4:** Antibiotic resistance patterns (%).

	AMP	CZ	AN	CN	FEP	CAZ	CRO	TZP	CIP	TGC	MER	IMP	COL	SXT	VAN	LZD
*E coli*					71.4	100	85.7	14.3	42.9	14.3	0	0	0	57.1		
*A baumannii*			75	100		100		100	100	0	100	100	0			
*E cloacae*										0	66.7	66.7	0	0		
*K pneumoniae*			0	0	50	50	0	50	50	0	0	0	0	50		
*P aeruginosa*			0	0	50	0	0	0	50		0	0	0			
*E faecalis*															0	0
*S aureus*	50	50												50	0	0
*M morganii*			0	0	0	0	0	0	0		0	0	100	100		

*A baumannii* = *Acinetobacter baumannii*, AMP = ampicillin, AN = amikacin, CAZ = ceftazidime, CIP = ciprofloxacin, CN = gentamicin, COL = polymyxin-E (colistin), CRO = ceftriaxone, CZ = cefazolin, *E cloacae* = *Enterobacter cloacae*, *E coli* = *Escherichia coli*, *E faecalis* = *Enterococcus faecalis*, FEP = cefepime, IMP = imipenem, *K pneumoniae* = *Klebsiella pneumonia*, LZD = linezolid, *M morganii* = *Morganella morganii*, MER = meropenem*, P aeruginosa* = *Pseudomonas aeruginosa, S aureus* = *Staphylococcus aureus,* SXT = trimethoprim-sulfamethoxazole, TGC = tigecycline, TZP = piperacillin-tazobactam, VAN = vancomycin.

## 4. Discussion

Infection development following arthroplasty leads to increased morbidity and mortality, extended hospitalization, and a significant financial burden.^[7]^ In a recent meta-analysis, Ramos et al reported an 11% mortality rate associated with PJI following THA, underscoring the critical need for a multidisciplinary approach to its management.^[8]^ The incidence of PJI post-TKA ranges from 0.5% to 2%; in contrast, the incidence after THA is reported to be between 0.5% to 1%.^[2,7,9]^ Compared with THA, TKA is associated with a higher risk of periprosthetic joint infection, likely due to thinner soft tissue coverage, increased mechanical stress during early mobilization, and the more superficial location of the knee joint, all of which may compromise wound healing and host defense. The larger contact surface area in knee prostheses also increases the risk of bacterial colonization.^[2,10]^ The identified risk factors for PJI include nonuse of ventilation filters in operating rooms, excess personnel traffic, improper sterilization of surgical materials, insufficient preoperative skin cleansing, male sex, smoking, RA, DM, depression, steroid use, and immunodeficiency.^[3,11]^ In our study, 83.3% of the participants were women and were predominantly affected by hip joint involvement (89%). All female patients with concomitant DM and OA were included in this study. Although 2 of our female patients were diagnosed with OA, there were also women who had not been diagnosed. Estrogen withdrawal after menopause increases the risk of OA and infection in women. DM also increases susceptibility to infections.^[12]^ We believe that the increased risk of THA in women is due to factors such as insufficient self-care in elderly women, poor nutrition, difficulties in accessing postoperative healthcare services compared to men, and a higher frequency of comorbidities in female patients. Wier et al demonstrated that irrespective of the surgeon’s individual experience, factors such as the hospital’s infrastructure, institutional expertise, standardized clinical protocols, and a multidisciplinary approach significantly contribute to lowering the risk of PJI.^[13]^ A linear-flow ventilation system was used in the operating rooms. We have difficulty limiting personnel traffic because we are training hospitals. Of these patients, 27.8% had DM and 11.1% had RA. The average age of the patients was 74 years, which was associated with immune disorders.

*P aeruginosa*, *S aureus*, and *Staphylococcus epidermidis* are capable of biofilm formation and adherence to prosthetic surfaces.^[3]^ Upon attachment, these bacteria proliferate and establish microcolonies, which are protected by the glycocalyx layer. Intercellular signaling within the biofilm further consolidates the matrix, rendering it highly antibiotic-resistant. Consequently, eradication of biofilms typically requires surgical intervention.

The diagnosis of PJI is challenging, necessitating the use of multiple markers in conjunction.^[14]^ Typically, our patients presented with joint pain, restricted mobility, and elevated local temperature. However, neither fever nor erythema are diagnostic. Although pain and limited movement are more sensitive indicators, they may also be observed in cases of aseptic loosening.^[15]^ The presence of a sinus tract related to the joint or prosthesis is the sole specific clinical finding for PJI, with concurrent bacteremia significantly aiding the diagnosis.^[16,17]^ In our cohort, a sinus tract was observed in 2 THA patients. The intraoperative discovery of purulent fluid around a prosthesis is indicative of infection but should not be considered conclusive for PJI.^[18]^ C-reactive protein (CRP), sedimentation, procalcitonin, hemogram, and radiography were requested from the patients, along with standard bacterial cultures.

An elevated CRP level, in the absence of other causes, is suggestive of PJI; however, normal CRP levels do not rule out this condition.^[19]^ Postoperatively, CRP levels typically normalizes within 2 to 3 weeks. Its levels may also be elevated in the context of advanced age, malignancy, RA, and other chronic illnesses, warranting the judicious use of this marker. In cases of acute PJI, CRP levels often exceed 100 mg/L. The erythrocyte sedimentation rate (ESR) normalized over approximately 3 months. A concurrent increase in both CRP and ESR enhances the diagnostic sensitivity of PJI.

Interleukin-6 (IL-6), which is secreted by monocytes and macrophages, has proven to be valuable in the diagnosis of PJI. A meta-analysis revealed that IL-6, when compared with CRP and ESR, demonstrated a higher sensitivity of 97% for detecting PJI.^[20]^

Counting white blood cells (WBC) and polymorphonuclear leukocytes (PNL) in synovial fluid is crucial for diagnosis. Synovial fluid aspiration is readily performed on the knee, whereas imaging methods guide the procedure on the hip. The analysis should include leukocyte count, leukocyte distribution, culture, and Gram staining of the synovial fluid. WBC counts >1500/µL and PNL presence ≥65% raise the suspicion of PJI. Values exceeding 3000 WBC/µL and 80% PNL strongly suggest PJI.^[21]^ However, leukocyte numbers may also be elevated in periprosthetic fractures, inflammatory arthritis, and metal allergies. The presence of leukocyte esterase in synovial fluid, which increases with leukocyte concentration, has been proposed as a rapid bedside test to support PJI diagnosis.^[22]^

CRP and IL-6 levels in synovial fluid have demonstrated increased sensitivity in diagnosing PJI compared to their serum counterparts.^[23,24]^ Alpha-defensin, primarily secreted by PNLs, acts as an endogenous pyrogen. Synovial alpha-defensin achieves high sensitivity and specificity for PJI diagnosis.^[25,26]^ Nevertheless, its use may lead to false-positive results in inflammatory diseases characterized by crystalloid accumulation within the joint.^[27]^

According to the diagnostic criteria for PJI published by the Musculoskeletal Infection Society in 2011, the presence of 2 major criteria or a combination of 4 minor criteria strongly supports PJI diagnosis (Table 5).

**Table 5:** Musculoskeletal infections society PJI diagnostic criteria.

2 major criteria	4/6 minor criteria
* Presence of sinus tract * Microbiological 2 culture positivity	* CRP and sedimentation elevation * Leukocyte increase in SF * PNL increase in SF * Presence of purulence * 1 microbiological culture positivity * Histology (≥5 PNL in ≥5 fields)

CRP = c-reactive protein, PNL = polymorphonuclear leukocyte, SF = synovial fluid.

The presence of intraoperative pus around a joint is a strong indicator of PJI; however, a similar presentation can also be seen in arthritis due to metal debris and crystalloid accumulation. Synovial fluid acquired by arthrocentesis is comparable in diagnostic yield to intraoperatively obtained fluid. In hemodynamically stable patients, antibiotics should be withheld for 2 weeks before sampling to enhance culture accuracy. For microbiological analysis, a minimum of 5 tissue samples should be collected intraoperatively from different areas of the interface membrane at the prosthesis–bone junction.^[28]^ Histologically, the presence of >5 PNLs per high-power field in these tissues, coupled with a single positive culture, is highly indicative of infection, particularly with Gram-negative bacilli, *S aureus*, and beta-hemolytic streptococci. In contrast, at least 2 positive cultures are generally required to establish the presence of low-virulence organisms such as *Cutibacterium acnes* and coagulase-negative staphylococci.^[14,29]^ Detection of the same microorganism in synovial fluid cultures obtained intraoperatively and arthrocentesis notably strengthens the diagnosis. However, negative intraoperative cultures do not definitively rule out infection.^[4]^ Furthermore, culturing synovial fluid in blood culture bottles has proven to be more sensitive than using solid agar or thioglycollate media.^[30]^ In the study by Indelli et al, the importance of using molecular methods such as polymerase chain reaction and next-generation sequencing, particularly in culture-negative PJIs, was emphasized.^[31]^

The sensitivity of direct radiography in distinguishing between aseptic and septic loosening is limited because of the similarities in their presentations. Diagnostically, a gap exceeding 2 mm between the bone and prosthesis, as well as lytic lesions in the bone, are indicative of infection. Ultrasonography can detect periprosthetic fluid collections, soft tissue swelling, and abscess formation, and is useful for guiding synovial fluid aspiration in PJIs following THA. Despite the artifacts caused by the presence of a metal prosthesis, computed tomography exhibits higher sensitivity than direct radiography, is capable of illustrating joint fluid and sinus tracts, soft tissue abscesses, erosions around the prosthesis, and bone loss, and can help in planning surgical intervention. Magnetic resonance imaging outperforms computed tomography in revealing soft tissue abscesses, infection, inflammation, and periosteal reactions, although its effectiveness is compromised by metal-induced artifacts. The most reliable imaging techniques are 3-phase bone scintigraphy and leukocyte scintigraphy.^[32,33]^

The 2-stage approach the most commonly adopted method for the treatment of PJI. In the first stage, infected components and cement are removed through meticulous debridement, while targeted antibiotic therapy commences based on culture susceptibilities. In cases where cultures are negative, empirical therapy is initiated, conforming to the local microbiota profiles. This was followed by an antibiotic-free interval. The second stage is undertaken once infection is excluded, both clinically and via laboratory tests, during patient monitoring.^[34]^ This stage involves repeated debridement and use of antibiotic-loaded cement. Because of the exothermic curing of polymethylmethacrylate, antibiotics that were not deactivated by heat were selected. These antibiotics should be bactericidal, capable of integration into cement, thermally stable, and water-soluble. Antibiotic powders, such as gentamicin, cefuroxime, erythromycin-colistin, and vancomycin, were chosen based on initial culture findings, often used alone or in combination.^[35,36]^ Subsequently, a new prosthesis is implanted.

The isolated microorganisms vary depending on the stage of PJI, which in turn informs the empirical treatment strategy.^[36]^ Early-onset PJI typically involves Gram-positive cocci, *S aureus*, anaerobic bacteria, or a polymicrobial etiology. Delayed PJI is more frequently associated with coagulase-negative staphylococci, low-virulence skin flora including *C acnes*, and enterococci.^[7]^ Gram-negative bacilli, *S aureus*, and beta-hemolytic streptococci are commonly identified pathogens.^[3,7,9,36]^ While many studies have reported a predominance of Gram-positive organisms, our study found that Gram-negative organisms were the prevailing pathogens, accounting for 83.4%. The gastrointestinal origin of these organisms is speculated to be due to barrier dysfunction, advanced age, and immunodeficiency; however, this has not been confirmed with concurrent blood cultures. Infection with rare microorganisms such as *Mycobacterium tuberculosis* and Pasteurella spp. has also been documented.^[37,38]^

In our study, intraoperative cultures yielded 24 microorganisms from 18 patients (6%), a prevalence that exceeded that reported in the literature.^[2,7]^ Females comprised 83.4% of our patient cohort, although there was variability in sex distribution across studies.^[39]^ The mean age of patients with PJI was 74 years. Aside from one 23-year-old patient who required total knee arthroplasty following a non-vehicular traffic accident, the remainder of the patients were of advanced age. The mean length of hospital stay was 46 days. Notably, all 16 patients who underwent THA presented femoral fractures. Two patients had sinus tract involvement. Since a single culture was obtained from the patients, none of them had 2 positive cultures. As all of them met the minor criteria, only 2 of our patients fit the Musculoskeletal Infection Society classification.

In our cohort, 87.5% of patients with PJIs had a peripheral venous catheter, 37.5% had a surgical drain, 4 patients had both a urinary and a central venous catheter, and one patient had a pericardial tube in situ. Catheter use has been associated with an increased risk of infection.^[40]^ Inadequate catheter care can increase the incidence of PJI originating from the skin flora, suggesting that catheters should be avoided when possible and promptly removed. Lopez-Contreras et al identified, coagulase-negative staphylococci and *S. aureus* as the most common pathogens.^[41]^ Notably, in our study, no PJIs were attributed to the skin flora. This may be due to the initiation of antibiotics before obtaining cultures when PJI is suspected, which can suppress bacterial growth, as well as our tendency to interpret coagulase-negative staphylococcal growth as contamination or colonization.

In this cohort, 5 patients (20.8%) required erythrocyte suspension transfusions. Causes included anemia in 3 patients and significant blood loss during surgery in 2. Addressing anemia prior to elective surgical procedures is critical for minimizing the risk of transfusion-related infections, whether bacterial, viral, or parasitic, as well as other associated complications.

In this study, 5 patients were administered H2 receptor antagonists, and one patient underwent intubation in the postoperative intensive care unit, with subsequent monitoring over a 3-day period.

Hypertension was the most common comorbidity in patients with PJIs (55.6%, followed by DM (27.8%). Anemia, congestive heart failure, and dementia were present in 3 patients each. Two patients were diagnosed with OA, chronic kidney disease, cerebrovascular disease, RA, atrial fibrillation, coronary artery disease, or chronic obstructive pulmonary disease. Additionally, individual patients presented with other conditions such as gout, Parkinson disease, renal transplantation, hyperthyroidism, chronic liver disease, asthma bronchiale, ventricular fibrillation, tricuspid regurgitation, myelodysplastic syndrome, delirium, benign prostatic hypertrophy, and acute myeloid leukemia (Table 1).^[7]^

A total of 66.7% of PJIs were classified as primary deep incisional, 4.2% as primary superficial incisional surgical site infections, and 29.2% as PJI. Of the isolated microorganisms, 83.4% were Gram-negative and the remainder were Gram-positive (Table 3). *E coli* was the most prevalent Gram-negative microorganism, constituting 29.2% of the isolates. Among the isolated *E coli* strains, over 50% were resistant to agents such as ceftriaxone, cefepime, ceftazidime, and trimethoprim-sulfamethoxazole. Tigecycline resistance was recorded in 14.3% of isolates, whereas resistance to colistin and carbapenems was not detected (Table 4).

*A baumannii* strains demonstrated sensitivity to tigecycline and colistin but exhibited resistance to all other tested antibiotics. The fact that *A baumannii* constituted 20.9% of the causative agents underscores the need for a critical review of standard isolation precautions and hand hygiene practices in our clinic.

*E cloacae* constituted 12.5% of the other enteric bacilli, while both *K pneumoniae* and *P aeruginosa* accounted for 8.3% each. *E cloacae* exhibited no resistance to tigecycline, colistin, or trimethoprim-sulfamethoxazole; however, carbapenem resistance was observed. In contrast, *K pneumoniae* and *P aeruginosa* showed a 50% resistance rate to both cefepime and ciprofloxacin but no resistance to colistin and carbapenems.

*M morganii* has been identified as the causative agent of PJI. It exhibited resistance to colistin and trimethoprim-sulfamethoxazole, yet remained sensitive to all other tested antibiotics.

*E faecalis* and *S aureus* were isolated in 2 cases of PJIs, accounting for 8.3% of the cases. All *E faecalis* strains demonstrated sensitivity to ampicillin, whereas one *S aureus* strain exhibited resistance to methicillin (Tables 3 and 4).

## 5. Conclusion

PJIs present a diagnostic challenge and often require prolonged treatment. Importantly, the prevention of PJIs is not only more straightforward but also significantly more cost-effective than treatment. Establishing laminar airflow in the operating room, limiting personnel traffic, implementing institution-specific infection prevention protocols, and providing feedback to staff can reduce the incidence of PJI. In cases where PJI is suspected, the evaluation should be guided by epidemiological findings and a thorough assessment of risk factors, supported by imaging studies and appropriate antibiotic management. Isolation of the causative microorganism is crucial for the initiation of effective parenteral therapy, followed by a sequential oral regimen. PJIs present a diagnostic challenge and often require prolonged treatment. Importantly, the prevention of PJIs is not only more straightforward but also significantly more cost-effective than treatment. Establishing laminar airflow in the operating room, limiting personnel traffic, implementing institution-specific infection prevention protocols, and providing feedback to staff can reduce the incidence of PJI. In cases where PJI is suspected, the evaluation should be guided by epidemiological findings and a thorough assessment of risk factors, supported by imaging studies and appropriate antibiotic management. Further studies are needed to more clearly define the diagnostic and preventive strategies.

## Acknowledgments

The authors acknowledge that they are responsible for the scientific content of the article in the stages of planning the study, collecting, analyzing, and interpreting the data, writing, scientifically reviewing the content, and approving the final version.

## Author contributions

**Conceptualization**: Bülent Kaya, Suzan Şahin, Serap Demir Tekol, Engin Eceviz.

**Data curation**: Bülent Kaya, Suzan Şahin, Serap Demir Tekol, Engin Eceviz.

**Formal analysis**: Bülent Kaya, Suzan Şahin, Serap Demir Tekol, Engin Eceviz.

**Funding acquisition**: Bülent Kaya, Suzan Şahin, Serap Demir Tekol, Engin Eceviz.

**Investigation**: Bülent Kaya, Suzan Şahin, Serap Demir Tekol, Engin Eceviz.

**Methodology**: Bülent Kaya, Suzan Şahin, Serap Demir Tekol, Engin Eceviz.

**Project administration**: Bülent Kaya, Suzan Şahin, Serap Demir Tekol, Engin Eceviz.

**Resources**: Bülent Kaya, Suzan Şahin, Serap Demir Tekol, Engin Eceviz.

**Software**: Bülent Kaya, Suzan Şahin, Serap Demir Tekol, Engin Eceviz.

**Supervision**: Bülent Kaya, Suzan Şahin, Serap Demir Tekol, Engin Eceviz.

**Validation**: Bülent Kaya, Suzan Şahin, Serap Demir Tekol, Engin Eceviz.

**Visualization**: Bülent Kaya, Suzan Şahin, Serap Demir Tekol, Engin Eceviz.

**Writing – original draft**: Bülent Kaya, Suzan Şahin, Serap Demir Tekol, Engin Eceviz.

**Writing – review & editing**: Bülent Kaya, Suzan Şahin, Serap Demir Tekol, Engin Eceviz.
